# The help-seeking process and predictors of mental health care use among individuals with depressive symptoms: a machine learning approach

**DOI:** 10.3389/fpubh.2024.1504720

**Published:** 2024-11-20

**Authors:** Vanessa Juergensen, Lina-Jolien Peter, David Steyrl, Cindy Sumaly Lor, Anh Phi Bui, Thomas McLaren, Holger Muehlan, Samuel Tomczyk, Silke Schmidt, Georg Schomerus

**Affiliations:** ^1^Department of Psychiatry and Psychotherapy, Medical Faculty, University Leipzig, Leipzig, Germany; ^2^Department of Cognition, Emotion, and Methods in Psychology, Faculty of Psychology, University of Vienna, Vienna, Austria; ^3^Department of Health and Prevention, Institute of Psychology, University of Greifswald, Greifswald, Germany; ^4^Department of Medicine, Health & Medical University Erfurt, Erfurt, Germany; ^5^Department of Psychiatry and Psychotherapy, University of Leipzig Medical Center, Leipzig, Germany

**Keywords:** help-seeking, depressive symptoms, mental illness stigma, machine learning, healthcare use

## Abstract

**Purpose:**

The goal of the study was to identify the most important influences on professional healthcare use of people with depressive symptoms. We incorporated findings from research areas of health behaviors, stigma, and motivation to predict the help-seeking process variables from a wide range of personal factors and attitudes.

**Methods:**

A sample of 1,368 adults with untreated depressive symptoms participated in an online survey with three-and six-month follow-ups. We conducted multiple linear regressions for (a) help-seeking attitudes, and (b) help-seeking intentions, and logistic regression for (c) help-seeking behavior with machine learning methods.

**Results:**

While self-stigma and treatment experience are important influences on help-seeking attitudes, complaint perception is relevant for intention. The best predictor for healthcare use remains the intention. Along the help-seeking process, we detected a shift of relevant factors from broader perceptions of mental illness and help-seeking to concrete suffering, i.e., subjective symptom perception.

**Conclusion:**

The results suggest a spectrum of influencing factors ranging from personal, self-determined factors to socially normalized factors. We discuss social influences on professional help-seeking and the use of combined public health programs and tailored help-seeking interventions.

**Clinical trial registration:**

German Clinical Trials Register (https://drks.de/search/en): Identifier DRKS00023557.

## Introduction

Despite growing awareness of depression among the general public in Germany (1), only about 40% of individuals with depression seek help from mental health professionals, MHP (2). This treatment gap (3, 4) has spurred substantial scientific endeavors to understand and enhance help-seeking behavior (5). However, there is a lack of sufficiently comprehensive models in this area. Previous studies on help-seeking have primarily focused on attitudes or intentions, and less frequently behavior (6, 7). Efforts to integrate diverse help-seeking conceptualizations are underway, aiming to amalgamate varied definitions into a unified framework (8).

This study examines three aspects of the help-seeking process for mental illness: attitudes, intentions, and behavior. We summarize theoretical and empirical findings on influences affecting these variables. And use a machine learning model to identify significant influences, barriers, and facilitators.

The help-seeking process begins with recognizing one’s complaints as signs of a mental illness (9, 10). According to Ajzen’s *Theory of Planned Behavior*, TPB (11) help-seeking is conceptualized via attitudes towards seeking help, perceived behavioral control, and subjective norms. These factors play a significant role in determining the intention to seek help, which is the strongest predictor of help-seeking behavior [e.g., (6, 12, 13)]. Compared to actual behavior, help-seeking intention can be predicted with more explained variance, and from a higher number of predictors [e.g., (6, 7)].

Personal attitudes are important influences, particularly for people with mild to moderate symptoms (14, 15). Other factors, such as structural influences, cultural imprints, and education, should also be acknowledged (16, 17), as well as new global developments like the Covid-19 pandemic (18, 19). Additionally, higher symptom severity, functional deficits, and previous treatment experiences increase the likelihood of help-seeking (20).

Yet, it’s crucial to recognize that intention does not always translate into seeking treatment (21), particularly due to the substantial barrier of stigma, as outlined by Link and Phelan (22). It involves labeling and separating individuals with mental illness, leading to stereotypes and discrimination. Public stereotypes towards mental illness and treatment may be internalized by affected individuals, leading not only to experiencing but also anticipation of discrimination. People often refrain from seeking professional help due to negative beliefs, viewing it as a sign of inadequacy, inferiority, or diminished self-esteem. The impact of stigma on help-seeking lacks consistent significance across studies (10), probably varying with depression severity (23). Moreover, public stigma seems to play a role in the act of seeking help (24), but the influences are inconsistent and focused on intention (25–27).

Further, a comparably young theory to explore behavior change is the *Self-Determination Theory,* SDT (28). As part of SDT, Deci and Ryan (28) categorized motivation on a spectrum of self-determination, ranging from autonomous motivation (self-determined regulated behavior) to controlled motivation (externally regulated behavior). Autonomous rather than controlled motivation correlates with behavior changes and improved health outcomes (29).

Various other factors impact the help-seeking process, encompassing beliefs about an illness, its conceptualization, causes, and consequences (30). Jorm et al. (31) highlighted that the general public’s knowledge about mental illnesses is relatively low compared to physical health issues. Since then, mental health literacy, i.e., the knowledge and competency regarding symptoms and treatment options, has been shown to have an overall positive impact on the help-seeking intention (10, 32). In addition, the idea that mental health and illness are on a spectrum leads to those continuum beliefs having an impact on health variables, e.g., increasing problem recognition (33).

Help-seeking intentions also depend on the perceived causes of mental health problems. A balanced biopsychosocial model is preferred (34), also because biomedical causes alone were found to be associated with higher stigma (35, 36). Self-efficacy had an influence on help-seeking for physical health (13). However, for mental healthcare-seeking, a task-specific consideration might be worthwhile, because inconsistent findings exist (37, 38). The belief in the capability to influence own life events might rather imply to “handle situations on one’s own” instead of enhancing healthcare use (39). Specific help-seeking self-efficacy can lead to improved communication with health care personnel and facilitate coping with treatment consequences (40).

Hence, it is important to be aware that the introduced predictors might interact. Stigma is negatively associated with continuum beliefs (41) and mental health literacy (42). Perceived stigma is negatively associated with autonomous motivation (43). Hence, to comprehend help-seeking of individuals with depressive symptoms, exploring a comprehensive subjective perspective encompassing diverse attitudinal variables could be crucial.

Methodwise, machine learning enables accurate predictions that take multiple factors into account (44). Complex data can be predicted, and models are not biased by interactions between the variables, the variable scales, or oversimplification of the models (45–47). It automatizes the process of building analytic models with the idea that an algorithm can learn from data to identify different patterns of information. Machine learning methods in mental health research are a recent development (48, 49), primarily focusing on predicting treatment outcomes (50, 51). In the field of health behavior, many studies focus on samples with diagnosed major depression (52), but determinants of help-seeking can differ substantially depending on treatment experiences (53) and current symptoms (54). Thus, it would be useful to study a sample consisting of individuals with depressive symptoms, but not necessarily with diagnosis, therefore without initiated help-seeking process and without ongoing therapy.

**Figure 1:**
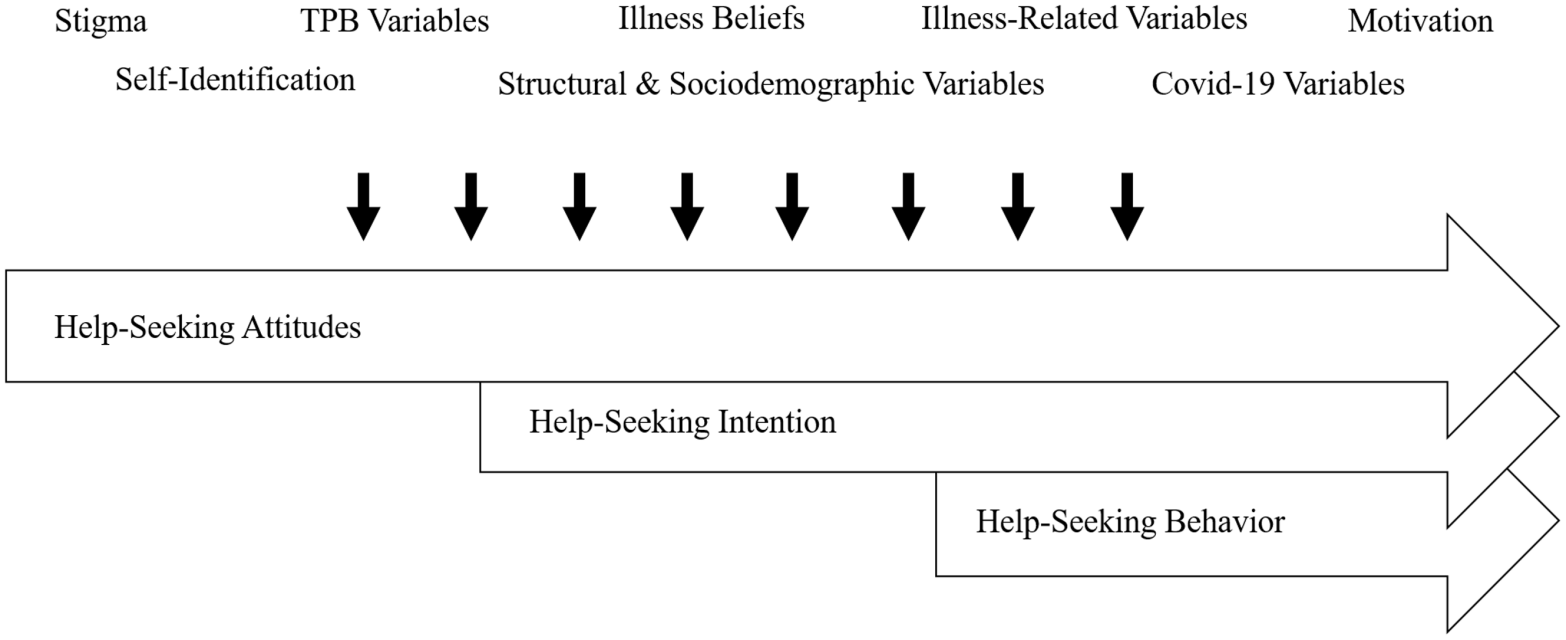
Hypothesized influences on the help-seeking process due to mental health problems, consisting of attitudes, intentions, and actual help-seeking behavior from mental health professionals (psychiatrist, psychologist, general practitioner, counselling center, neurologist). Hypothesized influences displayed as thematic categories. TPB variables: components of theory of planned behavior (11).

The aim for the current study is to identify predictors for (a) attitudes towards professional psychological help (*help-seeking attitudes*), (b) intentions to seek help from MHP (*help-seeking intentions*), and (c) prospective utilization of healthcare from MHP within three or six months (*help-seeking behavior*) among individuals currently experiencing untreated depressive symptoms. We incorporated a large set of potential predictors, mostly attitudinal, but also sociodemographic, and structural influences, derived from the theoretical and empirical research mentioned earlier. The comprehensive model is outlined in Figure 1. While replicating established findings, our primary objective is to consolidate results using machine learning methods, establishing a foundation of pertinent factors for help-seeking in future intervention studies. For an overview of hypothesized predictor variables for each outcome, consult Supplementary material S1–Predictor variables and measurements.

## Materials and methods

### Study design and participants

This study is part of a project about increasing healthcare use for people with depressive symptoms with a published study protocol (55), preregistration in the German Clinical Trial Register (DRKS00023557, 11.12.2020), and approval by the Ethics Commission of the University Medicine Greifswald (BB 061/18) and Leipzig (514/19-lk). The machine learning method was not initially included in the registration. Data collection (01/2021-09/2021) involved an online survey administered by the “respondi AG” online panel, they recruit and manage a pool of participants for online surveys or research studies. The panelists received invitations to participate in the study via email, as is the standard procedure of the online panel. To achieve the target sample size, a multiple of the required number of survey invitations was sent, based on the prevalence of depressive symptoms. Upon opening the survey, potential participants first viewed the study information and data security notice and provided informed consent by clicking the consent option in the respective selection menu. Participants were first presented with screening questions that assessed the presence and severity of depressive symptoms, as well as their utilization of therapy or treatment services. Participants with at least mild to moderate depressive symptom severity (PHQ-9 sum score of 8 or higher; 55) not currently undergoing treatment were included. Participants without the stated depressive symptoms, and/or who currently were undergoing treatment were not able to participate. Other than this, participants needed to be at least 18 years old and of sufficient German language skills, as provided through the panel registration of the participants. All participants who participated in the baseline survey were invited for follow-ups at three and six months via mail from the online panel again. For study enrollment and throughout exclusion of participants, see Supplementary Figure S1. For a comprehensive understanding of the study procedure, design, interventions, recruitment strategy, etc., consult the study protocol (55).

### Sample and power

The initial questionnaire was completed by *n* = 1867. As shown in Supplementary Figure S1, due to quality issues and questionable credibility, the following exclusions were made to comprise the final data: completion time under half the median study duration and monotone answer profiles (*n* = 116), PHQ-9 score < 8 in the second assessment 36 h later (*n* = 362), conflicting information about an individual’s sex between the study points (*n* = 12), and diverse sex, due to small sample (*n* = 9). The final sample size was *N* = 1,368.

Regarding power, large sample size is recommended because machine learning often involves multiple predictors. Precise sample size justification and power analysis for complex, high-dimensional, multivariable models from the machine learning field is still an open matter and no standards have been established (56). A disputed simple suggestion is that *N* = 50 is required to start any meaningful machine learning-based data analysis (57). Another suggestion is that 10 to 20 observations per degree of freedom (predictor) is reasonable (58). Based on these considerations we estimated a sufficient *N* for predictions for help-seeking attitudes, intention and behavior. For attitudes, we calculated with 50 independent features a required *N* of 500 to 1,000. For intention, we calculated with 55 independent features a required *N* of 505 to 1,100. For the behavior, we calculated with 63 independent features a required sample size of *N* of 640–1,260. We were able to perform machine learning models with *N* = 1,368 participants from baseline survey for help-seeking attitudes and intention and with *N* = 983 from follow-up for behavior.

### Measures

Supplementary Tables S1b,c provide an overview of all the measures and variables [refer to the study protocol for a complete list of measures including item examples (55)]. In the following, the dependent variables, help-seeking attitudes, intention, and behavior are described.

We assessed *help-seeking attitudes* with the attitudes toward seeking professional psychological help – Short form, ATSPPH-SF (59). Participants rated their agreement on 10 items about seeking help from a mental health professional (e.g., “Emotional difficulties like many things, tend to work out by themselves”) on a 4-point Likert scale from 1 = “strongly disagree” to 4 = “strongly agree.” In contrast to the TPB Attitude Scale, the ATSPPH-SF refers more to general attitudes towards help-seeking and not, like the TPB Attitude Scale, to subjective use and feelings of help-seeking in relation to actual reported symptoms. We consider help-seeking attitudes on the one hand as a dependent variable as well as an independent variable to predict help-seeking intentions and behavior on the other hand.

We measured *help-seeking intention* with a list of 15 persons/institutions to potentially seek help from Pescosolido and Boyer (60). Participants rated the likelihood of help-seeking for their mental health problems, respectively, on a 7-point Likert scale from 1 = “extremely unlikely” to 7 = “extremely likely.” Three factors of sources of help emerged from an explorative factor analysis. We operationalized each factor as a maximum score of the highest rated help-seeking intention. Only one of the three factors was used as dependent variable, it is called *intention to seek help from mental health professionals* and contained the following persons/institutions: psychologist, psychotherapist, psychiatrist, counselling center, general practitioner, and neurologist. This approach conceptualizes a rather broad range of healthcare services for mental health problems, allowing to capture mental healthcare infrastructure emphasizing external validity (2). The other two emerging factors were help-seeking intention from a *general professional* (as opposed to mental *health professional*) and contained the following persons/institutions: police, teacher, social worker, priest/pastor, fortune teller, alternative healer and *informal sources,* which contained the following persons: family, friends, colleagues. While help-seeking intention from mental health professionals will function as dependent variable, to be predicted, and as exogenous variable to predict help-seeking behavior, the other two forms of help-seeking intention (from general professionals and informal sources) will be considered as independent variables, as predictors for help-seeking intention from mental health professionals and for behavior.

We assessed *help-seeking behavior* three months and six months after the baseline with the same list described for help-seeking intention. Participants indicated whether they sought help in the past three months, respectively (0 = “no,” 1 = “yes”). If they answered affirmatively, they were asked whether the help sought was due to psychological complaints (1 = “yes, exclusively,” 2 = “yes, amongst other complaints,” 3 = “no, because of other complaints”). Thus, we coded answers as help-seeking behavior, if either “1” or “2” were stated. Questions for both follow-ups were collapsed into a dummy-coded variable of help-seeking within the last three or six months (*N* = 983) with 0 = “did not seek help for their psychological complaints” and 1 = “sought help for their psychological complaints by at least one of the follow-ups”.

### Analysis procedure

The machine learning framework was performed in Python v3.9.7 using scikit-learn library v1.0.2 and includes the following three parts: prediction model, generalizability testing, and model analysis (61). Using custom python scripts, three separate analyses were conducted: predicting help-seeking attitudes, intention, and behavior, respectively. ElasticNet (EN) linear models were employed to analyze attitudes and intentions related to help-seeking, while logistic regression (LR) models were used to examine help-seeking behavior.

EN applies L1-norm as well as L2-norm regularization to perform variable selection and counter multicollinearity in data (62, 63). The predictors of these analyses are listed in the Supplementary material S1. We expect collinearity to some degree since we included related aspects of stigma of mental health and help-seeking. LR models were chosen to perform the classification task of predicting if participants sought help within the last six month (0 = “did not seek help”; 1 = “sought help”). LR was also used in a regularized form (L1 and L2 penalties). To assess the models’ performance to unknown data—the generalizability of the models’ predictions to new data—a nested cross-validation (CV) procedure was employed (64). CV implements repeated splits of the data into training and testing sets. Nested CV is applied, to train a model where also the hyperparameters have to be optimized. The training data of the outer loop is used in an inner CV loop to do the hyperparameter evaluation. For help-seeking attitudes and help-seeking intentions, we applied a group-controlled shuffle-split scheme in the main (outer) CV loop, grouping by participants (20% of the participants in the testing set, 80% in the training set, 100 repetitions). For the nested (inner) CV procedure, we combined a shuffle-split scheme with a blended frugal cost optimization process [Microsoft FLAML, v1.0.0 (91)] to identify the best performing complexity parameters. For help-seeking behavior, we used the same schema, this time considering the time point. The complexity parameters that led to the highest prediction accuracy in the inner CV were subsequently used and all other parameters left at default settings, to train regressor models in the main CV loop. The models were subsequently tested on the respective testing set of the main CV loop. The testing set was explicitly not used in the inner CV loop.

Because the project on which this study is based contained interventions on illness beliefs that aimed to influence stigma and help-seeking, these interventions will be entered as control variables in a dummy-coded from (1 = received intervention; 0 = did not receive intervention). We do not expect them to have an influence on the connections assessed in this study, since all other variables entered are assessed pre-intervention.

Regression performance was measured with the prediction coefficient of determination, prediction *R^2^* (63). Prediction accuracy was used as measure for classification applied in LR. Notably, the prediction *R*^2^ will be smaller than *R*^2^ values of conventional statistical models because the prediction *R*^2^ measures prediction performance for unknown data and not *post hoc* model fit (63). Classification performance was measured with weighted classification accuracy, meaning that the unbalanced class in our sample, was up weighted so that the total contribution of each class was equal (63). Higher values indicate a better model fit. We assessed the importance of single predictors for the model’s performance of the linear regressions using the mean weights of the models (65). The absolute value of the weights that the models use to compute the predictions directly reflects the predictor’s importance (model feature importance). Hence the higher the absolute value of the weight of a predictor the more important it is. We assessed importance of single predictors for the model’s performance of the LR through calculating the odds ratio of the weights of the model (65).

Statistical significance of the prediction *R*^2^ metric as well as of the independent variables importance’s was assessed using a modified t-test that takes the sample dependence due to CV into account (66, 67). If necessary, t-test results were Bonferroni corrected for multiple comparisons.

## Results

### Participants

#### Models 1 and 2—help-seeking attitudes and intentions

The baseline sample consisted of *N* = 1,368 participants (*n* = 897 female, 65.6%). On average they were 42.38 years old (*SD* = 15.22, range 18–92), *n* = 717 (52.4%) had 12/13 years of schooling and *n* = 391 (29.7%) lived alone. The mean depression severity was 12.99 (*SD* = 4.21, range 8–27), indicating moderate severity (68). The mean subjective health status was 2.52 (*SD* = 0.81, range 1–5). Prior treatment due to mental health problems (including counseling, self-help groups, and online help) was reported by *n* = 644 (47.1%), *n* = 745 (54.5%) were aware of local services to seek help from, and *n* = 321 (23.5%) stated they “would not go to a doctor or therapist at the moment because of COVID-19.” A mean score of 2.80 was stated for help-seeking attitudes (*SD* = 0.56, range 1–5). The average maximum help-seeking intention on a scale from 1 to 7 was 4.26 (*SD* = 2.01) for MHP.

#### Model 3—help-seeking behavior

Within three or six months (*N* = 829), help from MHP was sought by *n* = 317 (32.2%), of which *n* = 239 reported help-seeking from a general practitioner, *n* = 119 from a psychologist, psychotherapist, or psychiatrist, and *n* = 52 from a counselor. *N* = 529 (53.8%) reported that they sought help from family, friends, or colleagues, and *n* = 42 (4.3%) sought help from general professionals (police, teacher, alternative healer, etc.). The mean age was 44.38 years (*SD* = 15.18), *n* = 513 (52.2%) had 12/13 years of schooling and *n* = 308 (31.9%) lived alone. Abbreviations for the pedictors used in the study can be found in the Supplementary material S2–Results. A correlation matrix of all included predictors and outcomes can also be found in the Supplementary Table S2a.

### Predictors of the help-seeking process

The regression models could explain 58% of the variance of *help-seeking* and 46% of the variance of *help-seeking intention*. The mean of the accuracy of the LR model on *help-seeking behavior* was 71.17. For detailed model performances, refer to Table 1.

**Table 1:** Model performances of the three regression models.

	*R^2^*_average_	*R^2^*_median_	*p*
Help-seeking attitudes	0.58	0.59	<0.001
Help-seeking intention	0.46	0.46	<0.001

	Accuracy_average_	Accuracy_median_	
Help-seeking behavior	71.17	71.31	<0.001

*R2 = prediction coefficient of determination. Accuracy =: prediction accuracy. p = p-value of significance, Bonferroni-adjusted. Significance p ≤ 0.05.*

**Figure 2:**
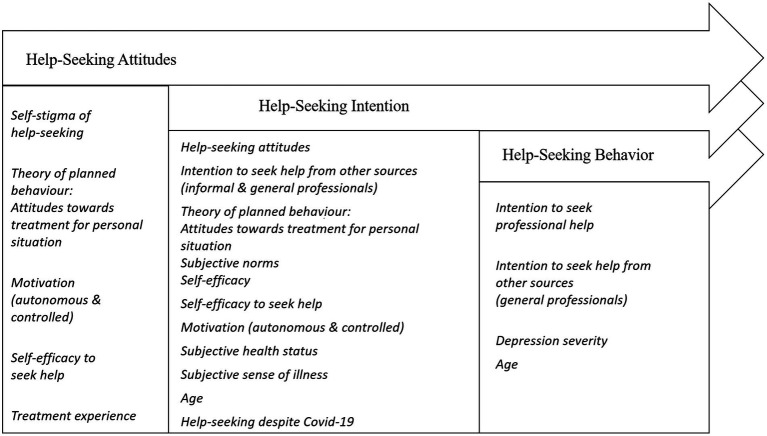
Significant influencing variables predicting help-seeking attitudes, intentions, and help-seeking behavior from mental health professionals based on the machine learning models. Only predictors with significant regression weights in the machine learning models are displayed under the respective dependent variable.

The significant predictors (Bonferroni-adjusted) which contributed to the overall explained variance together with their mean weight of the model-based feature importance can be seen in Table 2. Figure 2 provides an overview of the significant influencing variables.

**Table 2:** Help-seeking intention from mental health professionals: outcomes of en linear models (model-based feature importance) for help-seeking attitudes and intention from mental health professionals, outcomes of the LR model (model-based feature importance and odds ratio) for help-seeking behavior.

Predictors of help-seeking attitudes	*Mean Weight*	*p*
Autonomous motivation	0.20	< 0.001
Self-stigma of seeking help	−0.13	< 0.001
TPB—attitudes towards treatment for personal situation	0.09	< 0.001
Self-efficacy to seek professional help	0.04	< 0.001
Previous treatment experience (ref.: no treatment exp.)	0.04	< 0.001
Controlled motivation	0.04	< 0.001
Predictors of help-seeking intention from mental health professionals		
Help-seeking intention from general professionals	0.40	< 0.001
Help-seeking intention from informal sources	0.29	< 0.001
Help-seeking attitudes	0.28	< 0.001
Autonomous motivation	0.27	< 0.001
Age	0.27	< 0.001
TPB—subjective norms	0.18	< 0.001
Subjective sense of illness	0.14	< 0.001
Subjective health status	−0.13	< 0.001
Self-efficacy to seek professional help	0.14	< 0.001
COVID 19—potential help-seeking despite current pandemic (ref. not at all)	0.13	< 0.001
Controlled motivation	0.12	< 0.001
TPB– Self efficacy	0.08	< 0.001
COVID 19—no current potential help-seeking because pandemic (ref. not at all)	0.08	< 0.001

Predictors of help-seeking behavior	Mean weight	OR	*p*
Help-seeking intention from mental health professionals	0.26	1.30	< 0.001
Depression severity	0.06	1.06	< 0.001
Age	0.06	1.06	< 0.001
Help-seeking intention from general professionals	0.06	1.06	< 0.001

EN, elastic net. Prediction of help-seeking attitudes from a range of 50 variables, help-seeking intention (mental health professionals) from 55 variables, help-seeking behavior from 63 variables. *p* = *p*-value of significance, Bonferroni-adjusted. Significance *p* ≤ 0.05.

Higher autonomous motivation had the biggest influence on more positive help-seeking attitudes, while self-stigma of help-seeking had biggest and only negative influence. The intention to seek help from general professional or informal sources positively predicted the MHP help-seeking intention. More positive help-seeking attitudes, higher autonomous motivation, and higher age also had a moderate influence on help-seeking intention (MHP). Small significant influences were obtained from self-efficacy, subjective norms and controlled motivation, as well as from illness-related and pandemic-related variables.

The most important predictor for help-seeking behavior from MHP was the help-seeking intention from MHP with a moderate influence. Higher age, higher depression severity and intention to seek help from general professionals also increased the probability for participants to have sought help within the last three or six months (see Table 2).

See the Supplementary Table S2a and Supplementary Figures S2b–g for model-based feature importance of all predictors of EN and LR models and confusion matrix for LR model.

## Discussion

This study aims to identify factors influencing help-seeking among individuals currently experiencing untreated depressive symptoms. We examined variables influencing help-seeking, encompassing attitudes, intentions, and actual healthcare service use with machine learning models. The objective was to comprehend various determinants from different research streams and alleviate the treatment gap for individuals with depressive symptoms.

### Help-seeking attitudes

The significant impact of autonomous motivation on the help-seeking process for depressive symptoms expands the existing research because this connection has previously mainly been studied for physical health behaviors (69, 70), and only recently for mental health (71). Drawing from the results of this study, both aspects of motivation should be considered to explain attitudes towards help-seeking. People who report higher autonomous motivation to seek help tend to have generally more positive attitudes towards seeking professional help, while controlled motivation had a small positive influence as well.

Self-stigma of help-seeking can be seen as a barrier towards positive help-seeking attitudes, which corresponds to overall findings of the most prominent negative influences of internalized and treatment stigma on help-seeking (72). Nine other stigma measures did not show significant influences. It might be important to recognize that stigma still likely poses a major indirect barrier to the general help-seeking process, probably through a mediation effect (71). Help-seeking is not only directly influenced, but also indirectly by the attitudinal causes of influences, including stereotypes and public disadvantages (73).

This study moreover replicated results of treatment experiences altering help-seeking attitudes (74). Interestingly, self-efficacy to seek help positively influenced attitudes. This is in line with the idea of task-and context-specific self-efficacy referring to the confidence to know where and how to seek professional help as a differentiation from self-efficacy as confidence in helping oneself. Such a self-help self-efficacy seems to be a different construct without direct influence on help-seeking in this study, as well as in others (37). Seek help self-efficacy may resemble TPB’s behavioral control and merits nuanced future investigation (75).

### Help-seeking intention

The finding of higher intention to seek help from general professionals and informal sources (family, friends, colleagues) strongly influencing the MHP help-seeking intention suggests a general or mutually reinforcing intention when it comes to seeking help for mental health concerns. This aligns with Rüdell et al. (76) who identified a positive link between seeking community and professional help.

It further highlights the importance of social support for considering professional help for depression. Social context should be considered for tailored help-seeking interventions (77). These findings underscore the importance of emphasizing that there are different sources of help all of whom addressing the treatment gap. The predictive role of social norms is closely linked with factors like heightened awareness of social support and consideration of others’ opinions (7).

Moreover, the influences of positive help-seeking attitudes, social norms and self-efficacy as parts of TPB (11) replicate prior findings (6, 7). Seek-help self-efficacy was a significant feature again. The measurement of context-specific self-efficacy thus has demonstrated its’ usefulness and merits inclusion in future studies. Moreover, strengthening self-efficacy for help-seeking offers an approach to encourage contact with the health system. Combining self-efficacy and social surroundings as interventional approaches could be realized through fostering community self-efficacy (78), such as being aware of “your impact on the community” (p. 79).

Further, autonomous motivation had a higher influence on intention than controlled motivation, aligning with previous findings (29, 70). The application of motivation from SDT for mental illness help-seeking is a novel promising approach, indicating that potential help-seeking for mental health problems can be motivated by self-determined reasons, like wanting to get help as an important personal decision, as well as by external, less-self-determined reasons like felt expectance of others. Determinants of the effects are to be examined in future studies, i.e., who benefits from which kind of motivation depending on socioeconomic status, culture, stigma, or other context factors.

Hagger and colleagues (69, 79) established a link between autonomous and controlled motivation and aspects of TPB. Both theories suggest influences of personal drivers and attitudes on the one hand, and social norms on the other hand. While additional empirical evidence is required to confirm this connection, our data show moderate correlations of *r* ≥ 0.50 between the respective variables (see Supplement Table S2a). To seek help effectively, individuals may benefit from being part of a social network that encourages seeking help and being motivated by personal autonomy to increase positive attitudes and intentions towards seeking help. Concluding, attitudes and autonomous motivation, as well as social norms and controlled motivation work together in a complementary manner.

Stigma having no significant influence contrasts other studies (26). Other factors with no significant impact included illness beliefs such as mental health literacy, causal beliefs, and continuum beliefs. Research findings for these variables are inconsistent (10, 36). Differences could lie within the sample characteristics depending on factors like treatment experiences and access to healthcare (53). Moreover, stigma hindering the general help-seeking process (72) makes indirect influences likely.

Given the survey’s time alignment with the COVID-19 pandemic, we considered that participants’ willingness to seek help might be influenced by factors like restricted options and infection risk. Despite a general reluctance to seek help, there was a higher intention to seek help during the pandemic if individuals desired assistance. This suggests that the intention to seek help for mental health issues may be less affected by limited opportunities. However, the actual barriers preventing help-seeking should additionally be retrospectively assessed.

### Help-seeking behavior

In line with previous findings, help-seeking intention emerged as the strongest predictor (6, 7). However, the intention cannot one-to-one be transferred to behavior, reflecting the consistent intention-behavior gap (21). Moreover, our investigation did not include an examination of how formal and informal help-seeking behaviors interact with each other, as this was outside of the scope. It is important to consider how these two forms of help can be accessed simultaneously and still potentially influence each other (80). Future studies could investigate the relationship between seeking help from social surroundings and from professional sources.

Higher depressive symptoms linked to increased help-seeking probability, aligning with past findings on health status and functional impairment’s influence (14, 20, 80, 81). However, the aim should be to overcome the barriers as preventively as possible through activation-based interventions and to bring help-seeking within the scope of possibilities for people before they decide to do so because of their symptom severity.

Additionally, our research indicates that the likelihood of healthcare use increases with age, which aligns with previous studies on help-seeking behavior (52). This could be explained through the operationalization-wise decision to expand the definition of those seeking help to include general practitioners, leading to more frequent visits and increased chances to address mental health concerns as one ages. The findings consistently suggest the possibility of exploring impact factors on seeking help for specific age groups (82).

Some studies also report substantial sex differences for the probability of help-seeking (2, 83), which is not supported in our findings. However, sex differences are inconsistently found in other studies and conflicting findings exist (52). In our sample, men were intentionally recruited to represent sex differences in depression diagnoses (35% in the sample). This approach might have obscured sex differences, as it could attract men more interested in study participation, given their typical underrepresentation in mental-health online surveys.

Treatment experience emerged as a significant predictor for help-seeking attitudes but not for behavior, contrary to consistent findings in other studies (53). However, the coefficient was initially significant before correcting for *p*-values due to multiple testing. The impact of treatment experience on help-seeking might not have been as pronounced as in earlier research due to variations in how the concept was operationalized. Our study included general practitioners in help-seeking behavior, but participants’ responses about treatment experience likely referred to more specific psychological or psychiatric help, limiting construct congruence. Future studies could explore the quality of treatment experiences and participants’ coping with previous symptoms to gain a more comprehensive understanding of help-seeking. Such considerations can offer meaningful insights into the complex nature of help-seeking behavior and enhance the effectiveness of interventions promoting mental health and well-being.

### Strengths and limitations

This study examined help-seeking, including actual healthcare use among individuals with untreated depressive symptoms. Therefore, our results show high practical relevance as we aimed to investigate a sample that lacked initial access to healthcare and does not require symptoms to be diagnosed and labelled. Our models aimed to enhance understanding of attitudinal variables affecting help-seeking, though they may not be exhaustive. Notably, this study only addresses direct influences, neglecting potential indirect effects. Future studies should explore reciprocal models involving significant influences on help-seeking.

External factors like community elements, health insurance, somatic conditions, and ethnicity were not included in the analysis, contributing to potential inequalities. Moreover, intersectional inequalities through socio-economic position, gender, ethnicity, or sexual orientation were not addressed (84). It is crucial to examine the effects of both structural and external factors, which can create significant obstacles that public health initiatives should aim to address. Thus, a model incorporating the hereby-retrieved variables in combination with other external influences could be investigated by future studies as we did not collect information on cultural background nor ethnicity. It is important to note that the findings of this study may fail to generalize to other countries, or to minority groups (85) within the studied country. Moreover, people of diverse and non-binary gender have been excluded from the analysis due to small sample size. Many minority-specific factors are likely to have a prominent impact on help-seeking, including gaps in knowledge of health care providers (86). Underlining the importance of addressing these issues with further studies is crucial, given that some minority and gender diverse groups are less likely to receive treatment while being at a higher risk for developing certain mental health issues (87).

Unlike other studies (88), our approach to seeking help for mental health issues is more comprehensive. We operationalized help-seeking from psychotherapists, psychiatrists, and general practitioners, guided by exploratory factor analysis and the goal of assessing a broader spectrum of formal help sources (2). This strategy might have mitigated the impact of stigmatizing attitudes on the help-seeking process, as seeking treatment from psychiatrists is often negatively perceived by the public (73), while seeking help from a general practitioner is less stigmatized. Additionally, the intention subscales were operationalized as maximum scores, representing the highest intention towards one person or institution per subscale, without an overall probability of help-seeking across different institutions.

This study used an online panel for data collection as a cost-efficient and fast method allowing extensive reach and accessibility for a broad range of participants. However, these benefits are accompanied by limitations, such as potential data quality issues arising from the frequent participation of panelists in various studies, biases introduced by monetary incentives, and the self-selection of participants, which may compromise the representativeness of the sample. Consequently, we carefully considered exclusions of participants with questionable data quality, resulting in a smaller but more credible sample, although selection biases remain.

Machine learning has been proven to provide robust and replicable results (46) and is a valuable tool that enables the integration of multiple predictors into a single model (50). Given the current scenario marked by an exponential increase in studies, this strategy becomes especially relevant and timely, considering the growing number of variables for inclusion in investigations. While we endeavored to identify, replicate, and emphasize key factors influencing help-seeking, it’s essential to acknowledge the limitations of machine learning in data modelling. It does not imply causation, did not acknowledge interactions between the variables, which was endured in favor of the already complex model and for better interpretability, and diverse models can yield different results. Our use of model-based feature importance results in a complex model with robust predictions but challenges in interpretation (65).

Up to this point, only a minority of studies in the field investigated actual help-seeking behavior; therefore, we perceive this comprehensive examination of predictors as a basis for future studies. We decided to use an efficient binary measure to prospectively assess help-seeking behavior. To improve external validity of future studies, including more detailed assessment of variables characterizing help sought (quality factors, search process etc.) and more objective behavioral measures would be beneficial. One way could be to collaborate with medical care centers instead of relying on subjective assessments with biases of social desirability or self-selection, particularly for left-skewed stigma measures, and the potential bias in illness-related and help-seeking questions due to stigma. Moreover, it is important to consider internet and web-based help-seeking in future analyzes, which was beyond the scope of our study.

## Conclusion

We presented a comprehensive individual perspective on help-seeking of individuals with moderate depressive symptoms, including attitudinal variables based on multiple theoretical frameworks (52). An innovative machine learning approach reliably predicted help-seeking from a large number of variables. While help-seeking attitudes and intention could be predicted from multiple influences, rather few influences predicted behavior, with the intention to seek help explaining the most variance.

Help-seeking attitudes and intentions are influenced by rather attitudinal variables, on the one hand personal, autonomous attitudes and self-efficacy, on the other hand social norms and external influences. Less changeable variables such as depression severity and age become more influential on behavior as attitudinal variables lose direct influence.

As social norms seem to be important direct influences, future interventions might benefit from incorporating social strategies (89), like fostering support and emphasizing help-seeking-friendly social norms. Developing those interventions requires analyzing and including the various determinants of counteractive social norms and stigma. Effective anti-stigma messages are crucial, as stigma still is a complex barrier to help-seeking. We consider it a strength to investigate a population with symptoms of depression, who have not yet integrated into the healthcare system, although they may have an unclear need for assistance. Because our study found that illness perception and the severity of depressive symptoms predicted help-seeking behavior, future studies might benefit from paying even more attention to depression-specific decision-making processes (90).

Overall, this study highlights the complexity of the help-seeking process. Self-determined and social processes influence attitude and intention formation, while actual help-seeking is determined by already formed intention and symptom severity. Consequently, to effect changes in help-seeking behavior and improve the mental health of populations, an approach that considers the interaction between individual influencing factors and structural elements is necessary. Mental health care should be seen as a task for society as a whole, focusing on health determinants and social inequalities to reduce the number of people who need help and make access to help as convenient as possible for those who do.

## Data Availability

The raw data supporting the conclusions of this article will be made available by the authors, without undue reservation.
