# Supplementary material for: The help-seeking process and predictors of mental health care use among individuals with depressive symptoms: a machine learning approach
Source: Front Public Health. 2024 Nov 20;12:1504720. doi: 10.3389/fpubh.2024.1504720 (PMC11615672; doi:10.3389/fpubh.2024.1504720)
Supplement: Supplementary file 1 [file Supplementary_file_1.docx]

# The Help-Seeking Process and Predictors of Mental Health Care Use among Individuals with Depressive Symptoms: A Machine Learning Approach –

# Supplementary Material

Content

**S1 – Materials and Methods**

**Figure S1.** Flow Chart of Participation and Exclusions

**Table S1a.** Overview of Predictors for Each Machine Learning Model

**Table S1b.** Overview of the Measures used in the Current Study: Help-Seeking Process, Well-Being Variables and Illness Beliefs

**Table S1c.** Overview of the Measures used in the Current Study: Other Potential Influencing Variables

**S2 - Results**

**Abbreviations for the predictors used in the study**

**Table S2a.** Correlations of Important Predictors for the Three Models

**Figure S2b.** Model-Based Feature Importance for Help-Seeking Attitudes

**Figure S2c.** Elastic Net Regression Model for Help-Seeking Attitudes

**Figure S2d.** Model-Based Feature Importance for Help-Seeking Intention

**Figure S2e.** Elastic Net Regression Model for Help-seeking Intention

**Figure S2f.** Model-based Feature Importance for Help-seeking Behavior

**Figure S2g.** Confusion Matrix with Accuracy of the Logistic Regression Model with Help-Seeking Behavior as Outcome

S1 – Materials and Methods

**Figure S1**

*Flow Chart of Participation and Exclusions*


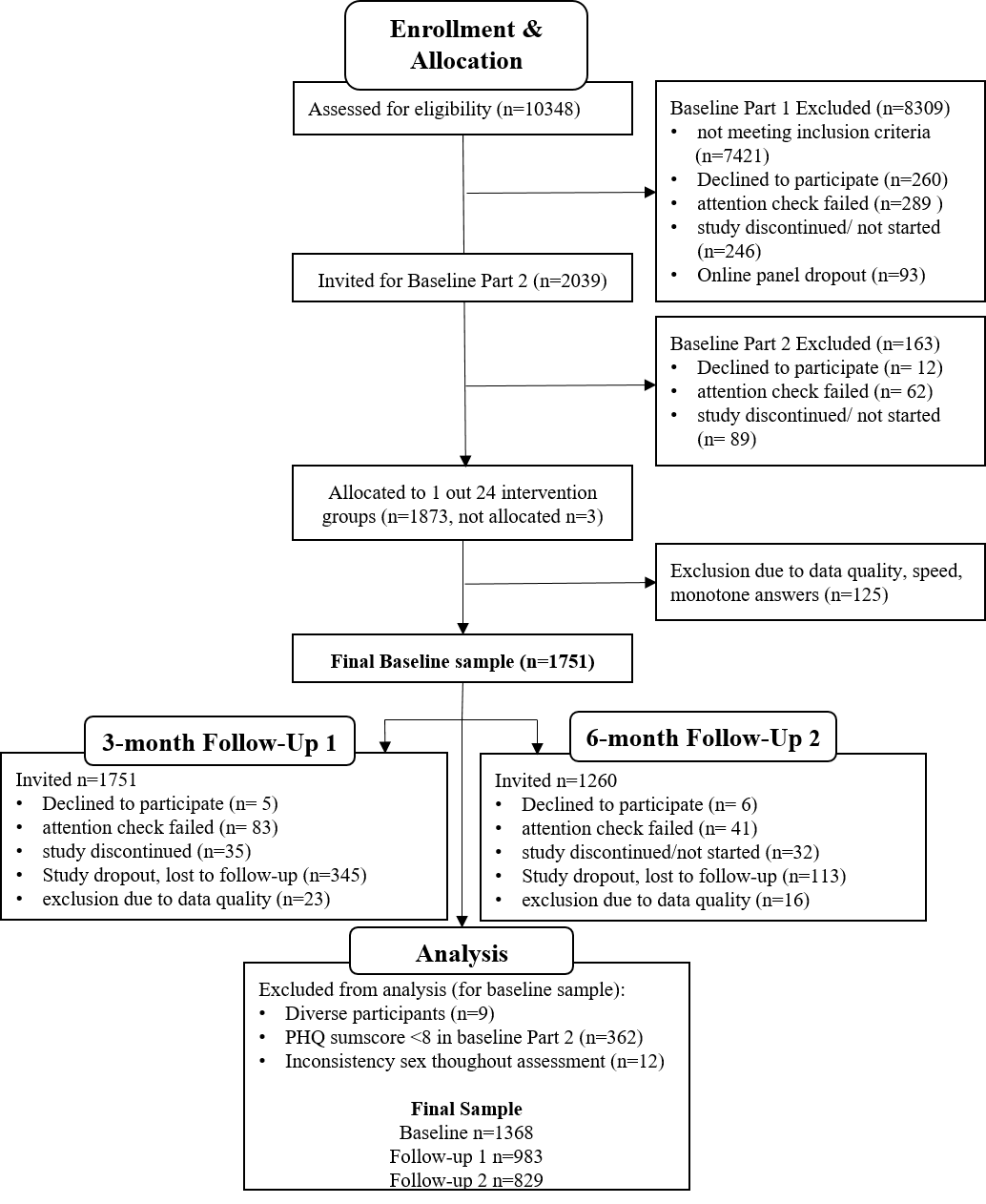


*Note.* Study consisted of the following online surveys: Baseline parts 1 & 2 (approx. one day apart), follow-up 1 after three months and follow-up 2 after six months. The Baseline survey contained interventions which are outside of the scope for this manuscript.

**Table S1a**

*Overview of Predictors for Each Machine Learning Model*

| **Help-Seeking Attitudes** | **Help-Seeking Intention** | **Help-Seeking Behavior** |
| --- | --- | --- |
|  |  | *Help-seeking attitudes* |
|  | *Help-seeking attitudes* | *Intention to seek professional help* |
|  | *Intention to seek help from other sources*  Informal  Non-professional | *Intention to seek help from other sources*  Informal  Non-professional |
| *Stigma* | *Stigma* | *Stigma* |
| *Self-Identification* | *Self-Identification* | *Self-Identification* |
| *TPB*  Attitudes  Subjective norms  Perceived controllability  Self-efficacy | *TPB*  Attitudes  Subjective norms  Perceived controllability  Self-efficacy | *TPB*  Attitudes  Subjective norms  Perceived controllability  Self-efficacy |
| *Intrapsychic*  Mental health literacy  Causal beliefs  Continuum beliefs  Self-efficacy seek help  Self-efficacy self help | *Intrapsychic*  Mental health literacy  Causal beliefs  Continuum beliefs  Self-efficacy seek help  Self-efficacy self help | *Intrapsychic*  Mental health literacy  Causal beliefs  Continuum beliefs  Self-efficacy seek help  Self-efficacy self help |
| *Motivation*  Autonomous  Controlled | *Motivation*  Autonomous  Controlled | *Motivation*  Autonomous  Controlled |
| *Illness-related*  Subjective sense of illness  Depression severity  Subjective health status  Quality of Life  Treatment experience | *Illness-related*  Subjective sense of illness  Depression severity  Subjective health status  Quality of Life  Treatment experience | *Illness-related*  Subjective sense of illness  Depression severity  Subjective health status  Quality of Life  Treatment experience |
| *Structural &*  *Sociodemographic*  Socioeconomic status  Age, Gender  Education  Income  Occupation  Service awareness  Household size  Personality traits | *Structural &*  *Sociodemographic*  Socioeconomic status  Age, Gender  Education  Income  Occupation  Service awareness  Household size  Personality traits | *Structural &*  *Sociodemographic*  Socioeconomic status  Age, Gender  Education  Income  Occupation  Service awareness  Household size  Personality traits |
| *Covid-19*  Help-seeking despite  pandemic  Distress  Rule compliance  Subjective restriction | *Covid-19*  Help-seeking despite  pandemic  Distress  Rule compliance  Subjective restriction | *Covid-19*  Help-seeking despite  pandemic  Distress  Rule compliance  Subjective restriction |

*Note.* While we surveyed the attitude and intention of those seeking help using a metric measure, we surveyed behavior after 3 months with a binary measure (yes/no).

**Table S1b**

*Overview of the Measures used in the Current Study: Help-Seeking Process, Well-Being Variables and Illness Beliefs*

| **Variable [reference]** | | | **Study name** | | | **Measurement (number of items)** | | | **Scoring (range)** | | ***α*** | |  |
| --- | --- | --- | --- | --- | --- | --- | --- | --- | --- | --- | --- | --- | --- |
| *Help-seeking process* | | | | | | | | | | | | |  |
| Help-seeking behavior after three or six months* [1] | | Help-seeking  behavior | | | List-wise assessment (15)  Subscale:  Mental health  professionals (MHP) | | | 0=no; 1=yes | | | | n.a. |  |
| Help-seeking intention*  [1] | | | Help-seeking intention (Mental health professionals/ General professionals/ Informal) | | | List-wise assessment (15); subscales | | | maximum scores (1-7) | |  | |  |
|  |  |  |  |  |  | Mental health professionals (MHP) | | |  |  | .91 | |  |
|  |  |  |  |  |  | General professionals | | |  |  | .81 | |  |
|  |  |  |  |  |  | Informal | | |  |  | .69 | |  |
| Attitude toward seeking professional psychological help*  [2] | | | Help-seeking attitudes | | | Attitude toward seeking professional psychological help-short form (10) | | | mean score  (1-4) | | .83 | |  |
| Self-identification as having a mental illness [3] | | | Self-identification | | | SELF-I (5) | | | mean score  (1-5) | | .90 | |  |
| Theory of planned behavior  [4] | | |  | | | Subscales | | |  | |  | |  |
|  |  |  | TPB - Attitudes towards treatment for personal situation | | | Attitudes towards treatment (3) | | | mean score  (1-2) | | .72 | |  |
|  |  |  | TPB - Subjective norms | | | Subjective norms (4) | | | mean score  (1-7) | | .90 | |  |
|  |  |  | TPB - Controllability | | | Perceived behavioral control - controllability (2) | | | mean score  (1-7) | | .82 | |  |
|  |  |  | TPB - Self-efficacy | | | Perceived behavioral control - self-efficacy (1) | | | 1-7 | | n.a. | |  |
| Motivation to seek help [5] | | |  | | | Autonomous and controlled motivation questionnaire, ACMQ subscales | | | mean scores  (1-5) | |  | |  |
|  |  |  | Autonomous motivation | | | Autonomous motivation (3) | | |  |  | .84 | |  |
|  |  |  | Controlled motivation | | | Controlled motivation (3) | | |  |  | .73 | |  |
| Previous treatment experience | | | Treatment experience | | | 1-item question | | | 1=experience; 0=no exp. | | n.a. | |  |
| *Symptoms and well-being* | | | | | | | | | | |  | | |
| Subjective sense of illness  [6] | | | Subjective sense of illness | | | Brief illness perception questionnaire (8) | | | mean score  (1-7) | | .66 | | |
| Subjective health status [7] | | | Subjective health status | | | SF-1 (1) | | | 1=bad - 5=excellent | | n.a. | | |
| Quality of life [8] | | | Quality of life | | | EUROHIS Quality of life (QOL)-index (8) | | | mean score  (1-5) | | .76 | | |
| Depression severity [9] | | | Depression severity | | | Patient health questionnaire (9) | | | sum score  (0-27) | | .70 | | |
| *Illness beliefs* | | | | | | | | | | | | | |
| Continuum beliefs | | | Continuum beliefs | | | Adapted from different instruments (9) | | | mean score,  (1-5) | | .68 | | |
| Mental health literacy  [10] | | | Mental health literacy | | | Depression literacy scale (12) | | | sum score of correct answers (range 0–12) | | .72 | | |
| Causal beliefs of mental illness [11] | | | Causal beliefs | | | Bio-psycho-social causal model (18) | | | index score (range 0–42.80) | | .85 | | |
| Self-efficacy to self-help  [12] | | | Self-efficacy to self help | | | Adapted items from the BRAHMS study (6) | | | mean score  (1-5) | | .80 | | |
| Self-efficacy to seek professional help [13] | | | Self-efficacy to seek professional help | | | Adapted items from healthcare use self-efficacy list (7) | | | mean score  (1-5) | | .86 | | |

*Notes.* Ranges: higher scores indicate higher agreement to variables. α = internal consistency (baseline measures). n.a. = not applicable. We assessed all measures at baseline, except for behavior (at follow-up). *** Variables and measurements explained in detail in the text.

**Table S1c**

*Overview of the Measures Used in the Current Study: other Potential Influencing Variables*

| **Variable [reference]** | | **Study name** | | **Measurement (number of items)** | | **Scoring (range)** | | ***α*** |  |
| --- | --- | --- | --- | --- | --- | --- | --- | --- | --- |
| *Stigmatizing attitudes* | | | | | | | | | |
| Stereotype awareness [14] | | Stereotype awareness | | Self-stigma of mental illness questionnaire, short-form (5) | | mean score  (1-5) | | .84 | |
| Stereotype agreement  [14] | | Stereotype agreement | | Self-stigma of mental illness questionnaire, short-form (5) | | mean score  (1-5) | | .80 | |
| Self-stigma of seeking help [15] | | Self-stigma of seeking help | | Short-form of the self-stigma for seeking help questionnaire (3) | | mean score  (1-5) | | .92 | |
| Desire of social distance from people with mental illness [16] | | Social distance | | Social distance scale, SDS (7) | | mean score  (1-5) | | .85 | |
| Agreement with the stereotype of blame [17] | | Blame | | Blame (3) | | mean score  (1-5) | | .79 | |
| Emotional reaction towards a person with a mental illness  [18] | |  | | ERMIS; subscales | | mean scores  (1-5) | |  | |
|  |  | Fear | | Fear (3) | |  |  | .82 | |
|  |  | Anger | | Anger (3) | |  |  | .80 | |
|  |  | Pro-social reactions | | Pro-social reactions (3) | |  |  | .64 | |
| Support for discrimination of persons with mental illness [19] | | Discrimination | | Discrimination (3) | | mean score  (1-5) | | .73 | |
| Shame for being mentally ill and seeking help [17] | | Shame | | Shame (2) | | mean score  (1-5) | | .86 | |
| *Sociodemography and biography* | | | | | | | | | |
| Age | | Age | | 1-item question | | ≥18, continuous | | n.a. | |
| Sex | | Sex | | 1-item question | | 1=female, 2=male | | n.a. | |
| Education | | Education | | years of schooling (1) | | dummy-coded (9,10,12+ years, other) | | n.a. | |
| Monthly income | | Income | | 1-item question | | dummy-coded (<1000€; -2500€;>2500€ | | n.a. | |
| Household size | | Household size | | number of 1-item question | | 1=single person household; 0=more than one | | n.a. | |
| Socioeconomic status  [20] | | SES | | SES Index for education, profession, income (score from sociodem. variables) | | 1=lowest; 6=highest | | n.a. | |
| Self-construal  [21] | | Self-construal- | | Subscales | | mean scores  (1-5) | |  | |
|  |  | Decisions | | Making decisions (6) | |  |  | .82 | |
|  |  | Self | | Looking after oneself (6) | |  |  | .76 | |
|  |  | Communicating | | Communicating with others (6) | |  |  | .78 | |
|  |  | Conflicting interests | | Dealing with conflicting interests (6) | |  |  | .73 | |
| *External factors* | | | | | | | | | |
| Covid-19 questions | | Covid-19 | | 4 items | |  | | n.a. | |
|  |  | Rule compliance | | Rule compliance | | 1-5 | |  |  |
|  |  | Restriction | | Restriction | |  |  |  |  |
|  |  | Psychological stress | | Psychological stress | |  |  |  |  |
|  |  | Potential help-seeking despite current pandemic | | Help-seeking despite pandemic (answers on item divided) | | 1= yes despite Covid; 0= not at all | |  |  |
|  |  | No current potential help-seeking because pandemic | | Help-seeking despite pandemic (answers on item divided) | | 1= no because of Covid; 0= not at all | |  |  |
| Perceived availability and accessibility to the health care system  [3] | | Service awareness | | Local service awareness | | 1=yes; 0=no | | n.a. | |

*Notes.* Ranges: higher scores indicate higher agreement to variables. α = internal consistency (baseline measures). n.a. = not applicable. We assessed all measures at baseline.

S2 - Results

**Abbreviations for the Predictors Used in the Study**

| **Name** | **Abbreviation** |
| --- | --- |
| Age | alter |
| Subjective health status | SF_1 |
| SES Education | SES_Bildung |
| SES Occupation | SES_Beruf |
| SES Income | SES_Einkommen |
| Illness perception | IPQR_Sum_Score |
| Depression severity | PHQ_Sum_Score |
| Self-identification of having a mental illness | SELFI_Mean_Score |
| Continuum Beliefs | CB_Mean_Score |
| Depression Literacy | DLIT_Sum_Score |
| Causal Beliefs | Ursachen_Score |
| Self-efficacy self help | Selbsthilfe_Mean_Score |
| Self-efficacy seek help | HUSEL_Mean_Score |
| Attitudes towards seeking professional help | ATSPPH_Mean_Score |
| TPB Subjective Norms | tpb_sn_Mean_Score |
| TPB Attitudes | tpb_att_Mean_Score |
| TPB Perceived behavior control | tpb_pbcontrol_Mean_Score |
| TPB Self efficacy | tpb_pbse |
| Autonomous motivation | ACMQ_auto_Mean_Score |
| Controlled Motivation | ACMQ_control_Mean_Score |
| Stereotype Awareness | SSMI_public_Mean_Score |
| Stereotype Agreement | SSMI_self_Mean_Score |
| Discrimination | Diskr_Mean_Score |
| Shame | Scham_Mean_Score |
| Social distance | SDS_Mean_Score |
| Blame | Schuld_Mean_Score |
| Self-stigma of seeking help | SSOSH_Mean_Score |
| Emotional reactions - Fear | ERMIS_angst_Mean_Score |
| Emotional reactions - Anger | ERMIS_wut_Mean_Score |
| Emotional reactions - Pro-Social Behavior | ERMIS_positiv_Mean_Score |
| Help-seeking intention-Professional | Max_Intention_MentalHealth |
| Help-Seeking Intention - General Professionals | Max_Intention_GenProf |
| Help-seeking intention - Informal | Max_Intention_Informal |
| Covid-19 - Compliance | covid_1 |
| Covid-19 - Restriction | covid_2 |
| Covid-19 - Psychological Stress | covid_3 |
| Quality of Life | QOL_Mean_Score |
| behav_alln | Help-Seeking Behavior |

**Table S2a**

*Correlations of Important Predictors for the Three Models*

|  | **ACMQ auto Mean Score** | **ACMQ control**  **Mean**  **Score** | **SSMI_**  **Public**  **Mean**  **Score** | **SSMI_self Mean**  **Score** | **DiskrMean**  **Score** | **SDS**  **Mean**  **Score** | **Scham Mean**  **Score** | **Schuld Mean**  **Score** | **SSOSH**  **Mean**  **Score** | **ERMIS**  **angst**  **Mean**  **Score** | **ERMIS**  **wut**  **Mean**  **Score** | **ERMIS_**  **positiv**  **Mean**  **Score** | **Max_**  **Intention**  **MentalHealth** | **Max_**  **Intention**  **GenProf** | **Max_**  **Intention**  **Informal** | **covid_1** | **covid_2** | **Covid**  **_3** | **QOL**  **Mean Score** | **behav_all** |
| --- | --- | --- | --- | --- | --- | --- | --- | --- | --- | --- | --- | --- | --- | --- | --- | --- | --- | --- | --- | --- |
| **alter** | -.06 | .03 | .13 | -.09 | -.01 | -.04 | .15 | -.01 | -.01 | .15 | .04 | -.04 | .11 | .04 | -.29 | .14 | -.05 | -.06 | -.10 | .15 |
| **SF_1** | -.01 | -.03 | .00 | .06 | .07 | .06 | -.03 | .08 | .04 | .05 | .00 | .02 | .14 | .04 | .17 | -.17 | .12 | .03 | **.49** | -.17 |
| **SES_Bildung** | -.04 | -.04 | -.01 | -.02 | -.01 | .04 | .06 | .06 | .03 | .05 | .04 | -.01 | -.06 | -.05 | .02 | .00 | .00 | -.06 | .20 | -.08 |
| **SES_Beruf** | .00 | .02 | -.02 | .05 | .03 | .06 | .06 | .11 | .11 | .05 | .08 | -.03 | -.01 | .02 | .07 | -.13 | .06 | .01 | .21 | .01 |
| **SES_Einkommen** | .01 | .04 | .03 | .00 | .05 | .05 | .12 | .07 | .05 | .07 | .09 | -.05 | .06 | .05 | -.02 | -.05 | .07 | .01 | .24 | .06 |
| **IPQR_Sum_Score** | .00 | .02 | .05 | .02 | -.05 | .02 | .03 | .06 | .01 | .07 | .02 | .06 | .05 | -.05 | -.13 | .05 | -.06 | .05 | **-.51** | .12 |
| **PHQ_Sum_Score** | .03 | .09 | .10 | .00 | -.06 | .05 | -.09 | .03 | .05 | -.02 | -.03 | .11 | .00 | -.03 | -.05 | .00 | -.04 | .10 | **-.48** | .14 |
| **SELFI_Mean_Score** | .27 | .06 | .08 | -.17 | **-.30** | -.18 | -.29 | -.26 | -.25 | -.17 | -.18 | .21 | .16 | -.05 | .04 | .07 | -.07 | .09 | **-.33** | .18 |
| **CB_Mean_Score** | .16 | -.16 | .03 | **-.38** | **-.36** | -.24 | **-.37** | **-.35** | -.29 | -.29 | -.29 | .14 | .03 | -.12 | .11 | .09 | -.05 | -.02 | .08 | .03 |
| **DLIT_Sum_Score** | .11 | -.16 | .01 | **-.32** | **-.33** | -.17 | -.25 | **-.37** | -.22 | -.18 | -.19 | .08 | .00 | -.18 | .03 | .11 | -.07 | -.04 | -.12 | .05 |
| **Ursachen_Score** | .09 | .17 | .07 | .19 | .12 | .06 | .06 | .14 | .04 | .13 | .17 | .04 | .09 | .11 | .01 | -.03 | -.03 | -.03 | -.04 | .03 |
| **Selbsthilfe_Mean**  **_Score** | .17 | .13 | -.11 | -.07 | .06 | -.12 | -.06 | .02 | -.15 | -.05 | .01 | .02 | .15 | .13 | .10 | .04 | .03 | .01 | .24 | .07 |
| **HUSEL_Mean_**  **Score** | .22 | .16 | .04 | -.05 | .11 | .23 | -.08 | -.01 | -.26 | -.07 | -.02 | .06 | .26 | .16 | .16 | .03 | .02 | -.02 | .29 | .11 |
| **ATSPPH_Mean_**  **Score** | **.68** | .29 | .01 | -.27 | -.19 | -.36 | -.24 | **.34** | **-.55** | -.14 | -.18 | .17 | **.49** | .12 | .25 | .13 | .02 | .09 | .06 | .25 |
| **tpb_sn_Mean_Score** | **.32** | **.54** | .05 | .03 | .01 | -.02 | -.13 | .02 | -.02 | .02 | .05 | .16 | **.38** | .22 | .23 | .00 | .02 | .12 | -.18 | .23 |
| **tpb_att_Mean_**  **Score** | **.62** | **.31** | .00 | -.18 | -.11 | -.15 | -.16 | -.16 | **-.30** | -.05 | -.08 | .11 | **.41** | .16 | .19 | .04 | .03 | .07 | .05 | .18 |
| **tpb_pbcontrol_**  **Mean_Score** | .05 | -.09 | -.06 | -.13 | -.04 | -.09 | -.09 | -.07 | -.06 | -.14 | -.14 | .00 | .02 | -.01 | .00 | -.01 | .03 | .04 | .16 | .03 |
| *Notes*. Reduced condensed variant of the most important predictors and their relationships to other predictors taken from the correlation matrix. Pearson correlation coefficients r. in bold: coefficients ≥ .30. Abbreviations of the variables can be found in the legend. | | | | | | | | | | | | | | | | | | | | |

**Figure S2b**


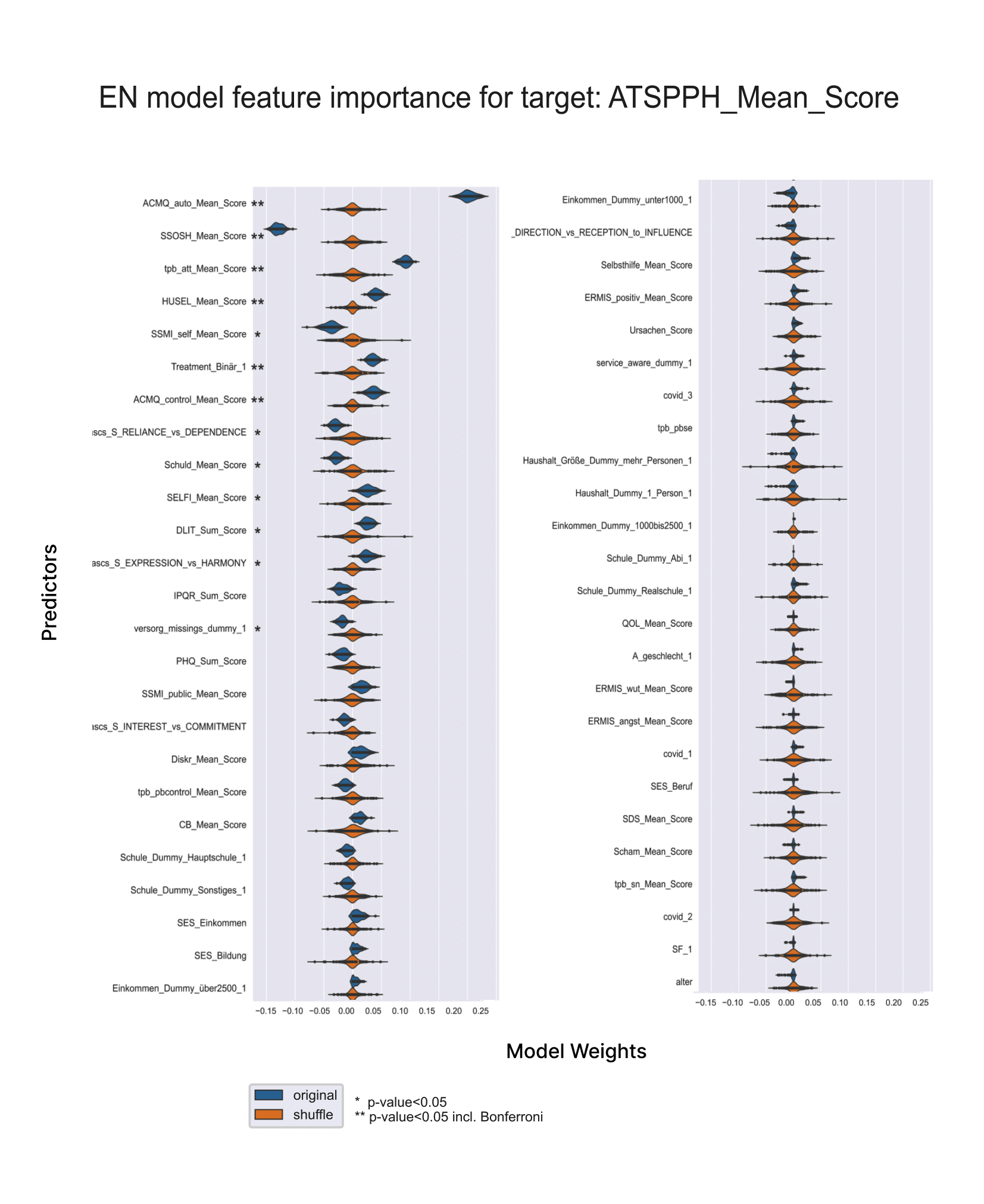
*Model-Based Feature Importance for Help-Seeking Attitudes*

*Note.* EN = Elastic Net. ATSPPH_Mean_Score = Attitudes towards seeking professional help. p-value of significance.

**Figure S2c**

*Elastic Net Regression Model for Help-Seeking Attitudes*


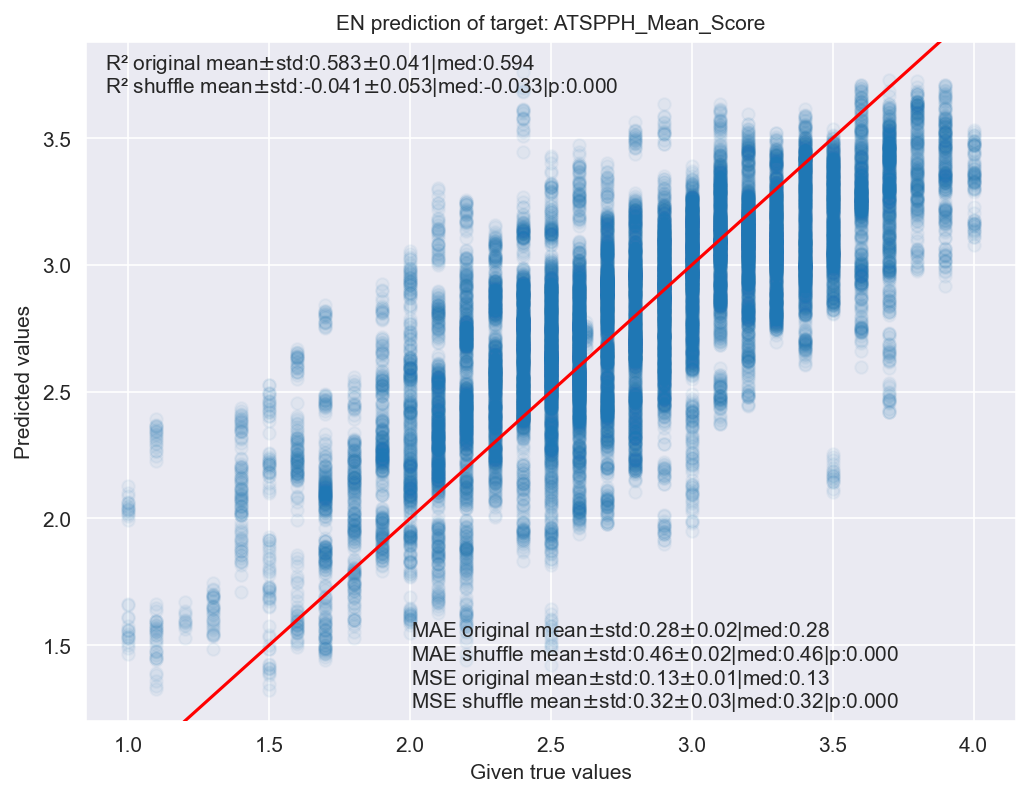


*Note.* EN = Elastic Net. ATSPPH_Mean_Score = Attitudes towards seeking professional help. *R² =* coefficient of determination*.* MAE = mean absolute error. MSE = mean standardized error. Std = standard deviation. med = median *p* = p-value of significance.

**Figure S2d**


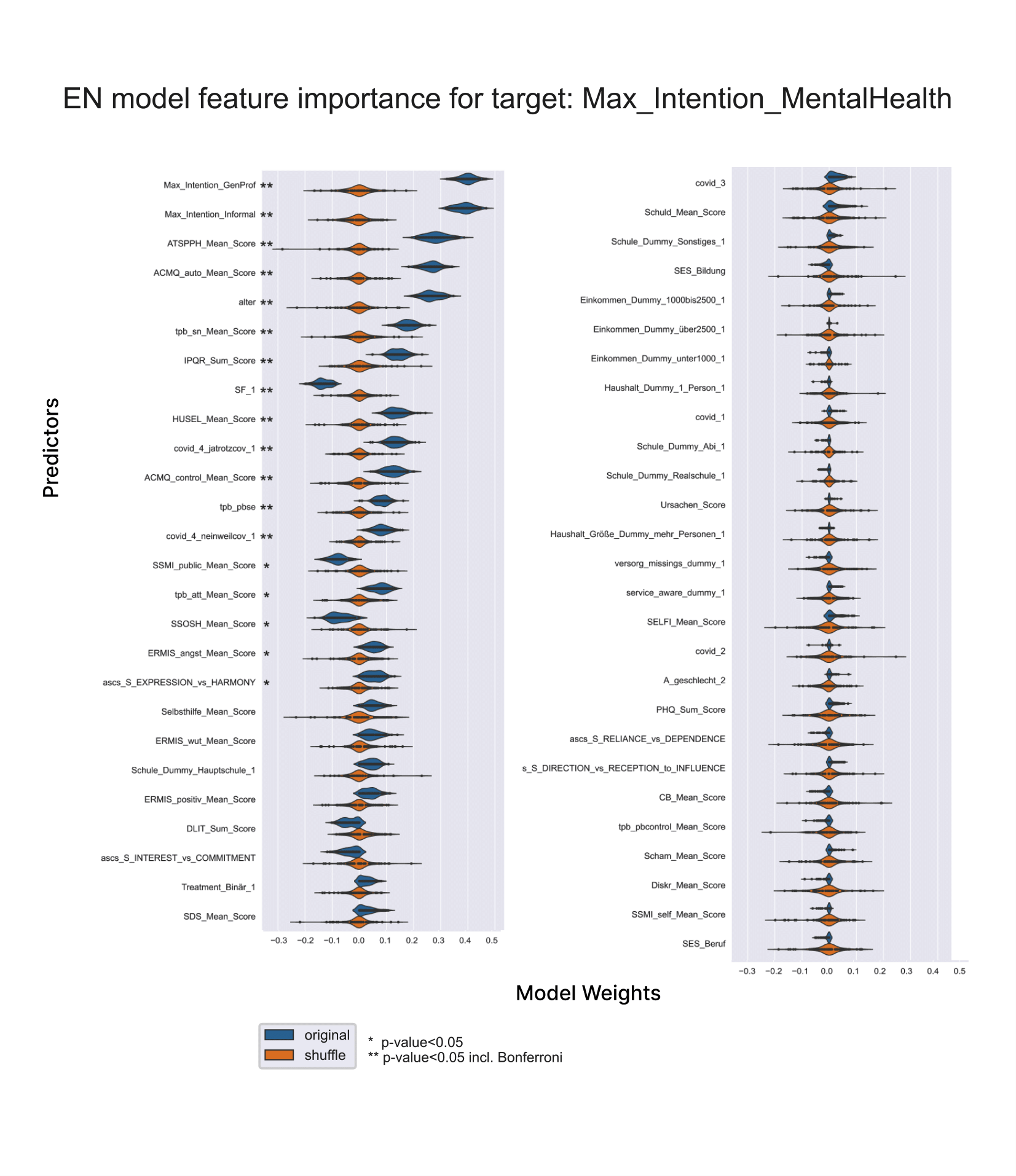
*Model-Based Feature Importance for Help-Seeking Intention*

*Note.* EN = Elastic Net. p-value of significance.

**Figure S2e**

*Elastic Net Regression Model for Help-seeking Intention*

.
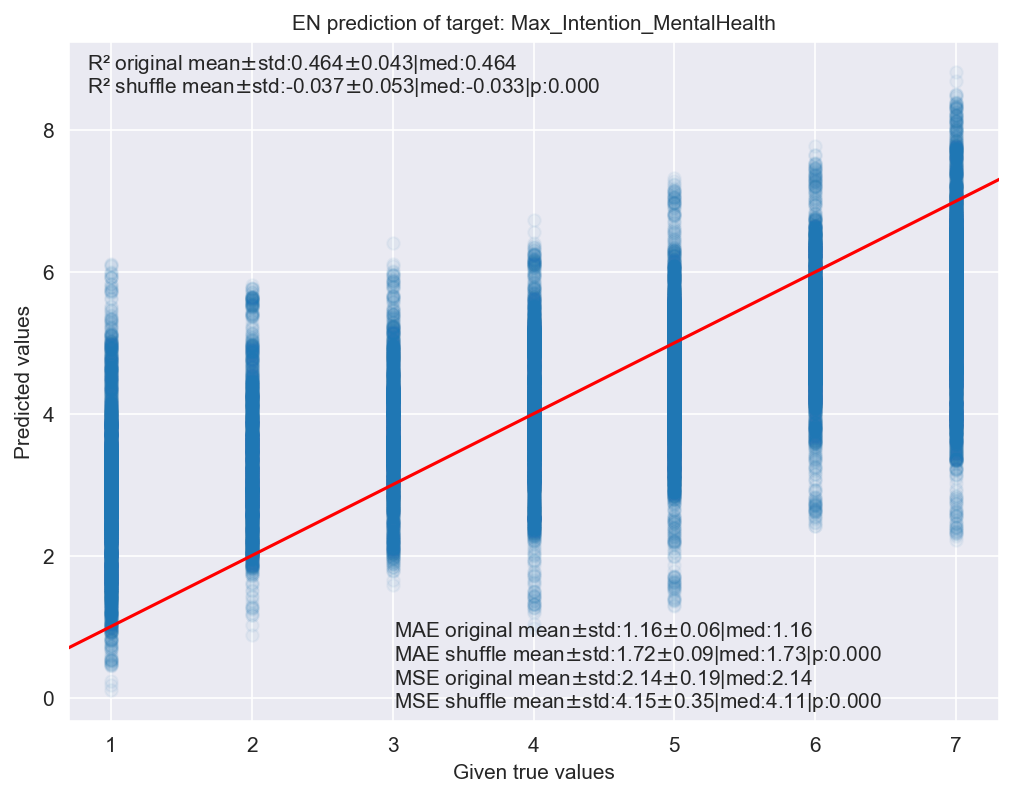


*Note.* EN = Elastic Net. *R² =* coefficient of determination*.* MAE = mean absolute error. MSE = mean standardized error. Std = standard deviation. med = median *p* = p-value of significance.

**Figure S2f**


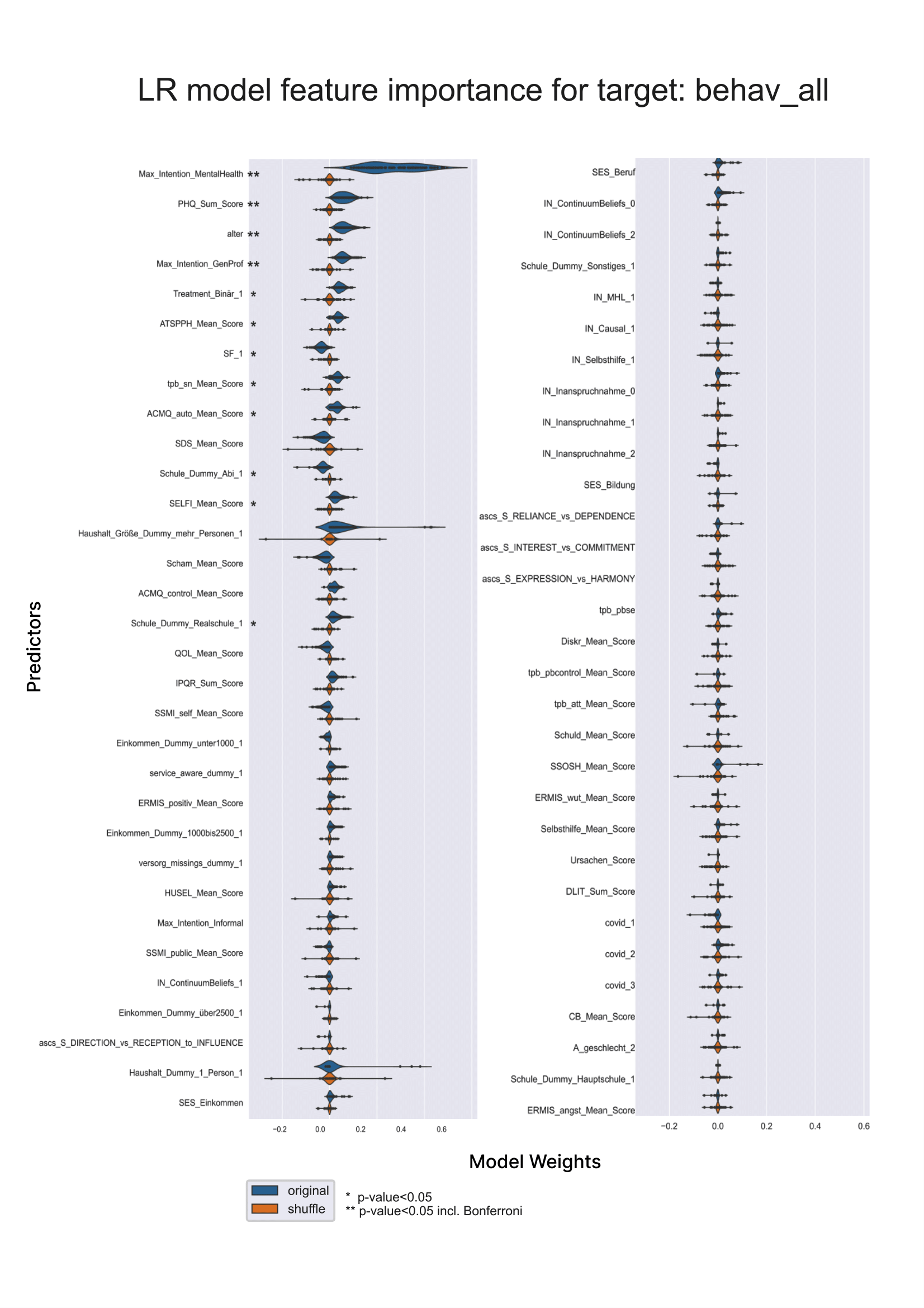
*Model-based Feature Importance for Help-seeking Behaviour*

*Note.* LR = Logistic Regression. p-value of significance.

**Figure S2g**

*Confusion Matrix with Accuracy of the Logistic Regression Model with Help-Seeking Behaviour as Outcome*


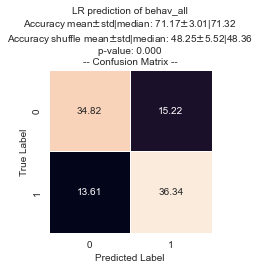


*Note.* LR = Logistic Regression Model. Bevah_all = Help-Seeking Behaviour. 0 = No help sought within the last three or six months from mental health professionals. 1 = Help sought within the last three or six months from mental health professionals. Std = standard deviation. p-value of significance.

References

1. Pescosolido BA, Boyer CA (2010) Understanding the context and dynamic social processes of mental health treatment. In: Scheid TL, Brown TN (eds) A handbook for the study of mental health: Social contexts, theories, and systems, Second edition. Cambridge University Press, Cambridge, pp 420–438

2. Fischer EH, Farina A (1995) Attitudes toward seeking professional psychologial help: A shortened form and considerations for research. Journal of College Student Development

3. Schomerus G, Muehlan H, Auer C et al. (2019) Validity and psychometric properties of the Self-Identification as Having a Mental Illness Scale (SELF-I) among currently untreated persons with mental health problems. Psychiatry Res 273:303–308. <https://doi.org/10.1016/j.psychres.2019.01.054>

4. Tomczyk S, Schomerus G, Stolzenburg S et al. (2020) Ready, Willing and Able? An Investigation of the Theory of Planned Behaviour in Help-Seeking for a Community Sample with Current Untreated Depressive Symptoms. Prev Sci 21:749–760. <https://doi.org/10.1007/s11121-020-01099-2>

5. Keleher B (2020) Evaluating the Effect of Basic Psychological Needs Support and Thwarting on Motivation for Emotionally Demanding Behaviour Change. UW Space

6. Broadbent E, Petrie KJ, Main J et al. (2006) The brief illness perception questionnaire. J Psychosom Res 60:631–637. <https://doi.org/10.1016/j.jpsychores.2005.10.020>

7. Morfeld M, Bullinger M, Kirchberger I (2011) Fragebogen zum Gesundheitszustand: SF-36 ; deutsche Version des Short Form-36 Health Survey, 2., erg. u. überarb. Aufl. Hogrefe, Göttingen, Wien u.a.

8. Brähler E, Muehlan H, Albani C et al. (2007) Teststatistische Prüfung und Normierung der deutschen Versionen des EUROHIS-QOL Lebensqualität-Index und des WHO-5 Wohlbefindens-Index. Diagnostica 53:83–96. <https://doi.org/10.1026/0012-1924.53.2.83>

9. Kroenke K, Spitzer RL, Williams JBW et al. (2010) The Patient Health Questionnaire Somatic, Anxiety, and Depressive Symptom Scales: a systematic review. Gen Hosp Psychiatry 32:345–359. <https://doi.org/10.1016/j.genhosppsych.2010.03.006>

10. Freitag S, Stolzenburg S, Schomerus G et al. (2018) Depressionswissen – Deutsche Übersetzung und Testung der Depression Literacy Scale (Depression Literacy - German Translation and Testing of the Depression Literacy Scale). Psychiatr Prax 45:412–419. <https://doi.org/10.1055/s-0043-119245>

11. Stolzenburg S, Freitag S, Schmidt S et al. (2018) Associations between causal attributions and personal stigmatizing attitudes in untreated persons with current mental health problems. Psychiatry Res 260:24–29. <https://doi.org/10.1016/j.psychres.2017.11.014>

12. Renner B, Hahn A, Schwarzer R (1996) Berlin Risk Appraisal and Health Motivation Study - Messinstrumente

13. Israel JI (2017) Healthcare use self-efficacy in adults who are homeless. Dissertation Abstracts International: Section B: The Sciences and Engineering

14. Corrigan PW, Michaels PJ, Vega E et al. (2012) Self-stigma of mental illness scale—short form: reliability and validity. Psychiatry Res 199:65–69. <https://doi.org/10.1016/j.psychres.2012.04.009>

15. Brenner RE, Colvin KF, Hammer JH et al. (2020) Using Item Response Theory to Develop Revised (SSOSH-7) and Ultra-Brief (SSOSH-3) Self-Stigma of Seeking Help Scales. Assessment:1073191120958496. <https://doi.org/10.1177/1073191120958496>

16. Angermeyer MC, Matschinger H (1997) Social distance towards the mentally ill: results of representative surveys in the Federal Republic of Germany. Psychol Med 27:131–141. <https://doi.org/10.1017/S0033291796004205>

17. Schomerus G, Angermeyer MC, Baumeister SE et al. (2016) An online intervention using information on the mental health-mental illness continuum to reduce stigma. Eur Psychiatry 32:21–27. <https://doi.org/10.1016/j.eurpsy.2015.11.006>

18. Angermeyer MC, Holzinger A, Matschinger H (2010) Emotional reactions to people with mental illness. Epidemiol Psichiatr Soc 19:26–32. <https://doi.org/10.1017/s1121189x00001573>

19. Schomerus G, Stolzenburg S, Freitag S et al. (2019) Stigma as a barrier to recognizing personal mental illness and seeking help: a prospective study among untreated persons with mental illness. Eur Arch Psychiatry Clin Neurosci 269:469–479. <https://doi.org/10.1007/s00406-018-0896-0>

20. Lampert T, Kroll L, Müters S et al. (2013) Messung des sozioökonomischen Status in der Studie zur Gesundheit Erwachsener in Deutschland (DEGS1) (Measurement of socioeconomic status in the German Health Interview and Examination Survey for Adults (DEGS1)). Bundesgesundheitsblatt Gesundheitsforschung Gesundheitsschutz 56:631–636. <https://doi.org/10.1007/s00103-012-1663-4>

21. Vignoles VL, Owe E, Becker M et al. (2016) Beyond the ‚east-west‘ dichotomy: Global variation in cultural models of selfhood. Journal of Experimental Psychology: General:966–1000. <https://doi.org/10.1037/xge0000175>

22. Nübel J, Müllender S, Hapke U et al. (2019) Epidemie der Depression? : Prävalenzentwicklung und Inanspruchnahme von Hilfs- und Versorgungsangeboten (Epidemic of depression? : Development of prevalence and help-seeking behaviour). Nervenarzt 90:1177–1186. <https://doi.org/10.1007/s00115-019-0681-y>

23. Rössler P (2017) Kumulierte Evidenzen: Replikationsstudien in der empirischen Kommunikationsforschung. Springer Fachmedien Wiesbaden, Wiesbaden

24. Rosenbusch H, Soldner F, Evans AM et al. (2021) Supervised machine learning methods in psychology: A practical introduction with annotated R code. Social and Personality Psychology Compass 15. <https://doi.org/10.1111/spc3.12579>

25. Lakens D (2021) Sample Size Justification

26. Vittinghoff E, McCulloch CE (2007) Relaxing the rule of ten events per variable in logistic and Cox regression. Am J Epidemiol 165:710–718

27. Friedman J, Hastie T, Tibshirani R (2010) Regularization Paths for Generalized Linear Models via Coordinate Descent. J Stat Soft 33:1–22

28. Hastie T, Tibshirani R, Friedman JH (2009) The elements of statistical learning: data mining, inference, and prediction, 2nd edn. Springer, New York

29. Cawley GC, Talbot N (2010) On Over-fitting in Model Selection and Subsequent Selection Bias in Performance Evaluation. Journal of Machine Learning Research:2079–2107

30. Wang C, Wu Q, Weimer M et al. (2021) Flaml: A fast and lightweight automl library. Proceedings of Machine Learning and Systems:434–447

31. Molnar C (2019) Interpretable machine learning: A guide for making black box models explainable. Leanpub

32. Nadeau C, Bengio Y (2003) Inference for the Generalization Error. Machine Learning 52:239–281. <https://doi.org/10.1023/A:1024068626366>

33. Bouckaert RR, Frank E (2004) Evaluating the Replicability of Significance Tests for Comparing Learning Algorithms. In: Kanade T, Kittler J, Kleinberg JM et al. (eds) Advances in Knowledge Discovery and Data Mining, vol 3056. Springer Berlin Heidelberg, Berlin, Heidelberg, pp 3–12
